# An interpretable and versatile machine learning approach for oocyte phenotyping

**DOI:** 10.1242/jcs.260281

**Published:** 2022-07-13

**Authors:** Gaelle Letort, Adrien Eichmuller, Christelle Da Silva, Elvira Nikalayevich, Flora Crozet, Jeremy Salle, Nicolas Minc, Elsa Labrune, Jean-Philippe Wolf, Marie-Emilie Terret, Marie-Hélène Verlhac

**Affiliations:** 1 Center for Interdisciplinary Research in Biology (CIRB), College de France, CNRS, INSERM, Université PSL, 75231 Paris, France; 2 Université Paris Cité, CNRS, Institut Jacques Monod, 75013 Paris, France; 3 Service de Médecine de la Reproduction, Hôpital Femme Mère Enfant, Hospices Civils de Lyon, 69500 Bron, France; 4 Université Claude Bernard Lyon 1, 69100 Lyon, France; 5 INSERM U1208, StemGamE, 69500 Bron, France; 6 Team ‘From Gametes To Birth’, Département Développement, Reproduction, Cancer, Institut Cochin, Inserm U1016, CNRS UMR8104, Université de Paris, 22 rue Mechain, 75014 Paris, France; 7 Service d'Histologie-Embryologie-Biologie de la Reproduction, Hôpital Cochin, Assistance Publique-Hôpitaux de Paris, 75014 Paris, France

**Keywords:** Characterization, Machine learning, Maturation, Oocyte, Segmentation

## Abstract

Meiotic maturation is a crucial step of oocyte formation, allowing its potential fertilization and embryo development. Elucidating this process is important for both fundamental research and assisted reproductive technology. However, few computational tools based on non-invasive measurements are available to characterize oocyte meiotic maturation. Here, we develop a computational framework to phenotype oocytes based on images acquired in transmitted light. We trained neural networks to segment the contour of oocytes and their zona pellucida using oocytes from diverse species. We defined a comprehensive set of morphological features to describe an oocyte. These steps were implemented in an open-source Fiji plugin. We present a feature-based machine learning pipeline to recognize oocyte populations and determine morphological differences between them. We first demonstrate its potential to screen oocytes from different strains and automatically identify their morphological characteristics. Its second application is to predict and characterize the maturation potential of oocytes. We identify the texture of the zona pellucida and cytoplasmic particle size as features to assess mouse oocyte maturation potential and tested whether these features were applicable to the developmental potential of human oocytes.

This article has an associated First Person interview with the first author of the paper.

## Abstract (precis)

**Summary:** Using machine learning, we have developed a computational framework, based on non-invasive transmitted light image, to phenotype and also predict the fate of oocytes coming from different species.

## INTRODUCTION

**Fig. 1.**
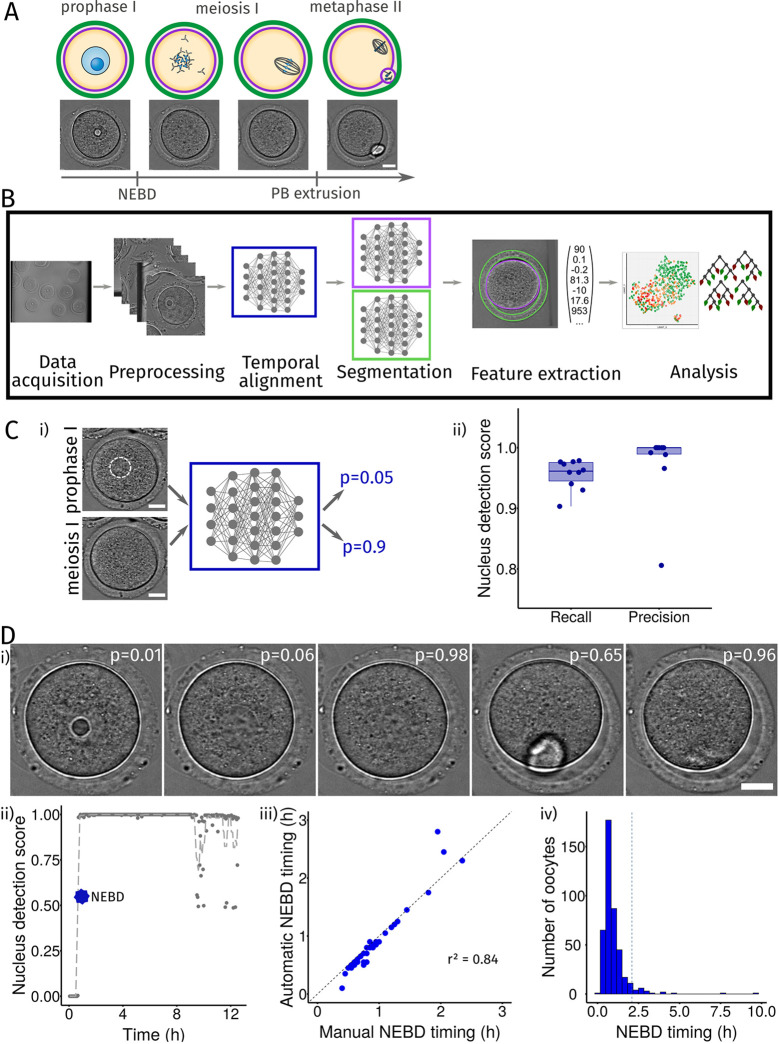
**A machine learning pipeline to characterize oocyte developmental potential.** (A) Scheme of the main steps of oocyte maturation, from prophase I to metaphase II. Schematic representation (top) and still images from a movie of a mouse maturing oocyte in transmitted light (bottom). Scale bar: 20 µm. Nuclear envelope breakdown, NEBD; polar body, PB. DNA is in blue, microtubules in dark gray, oocyte plasma membrane in purple, zona pellucida in green. (B) Scheme of the machine learning pipeline. Steps used in our pipeline: data acquisition, preprocessing, temporal alignment, segmentation, feature extraction and analysis. (C) Automatic nucleus detection from transmitted light images. (i) Training of the neural network (blue box) to recognize oocytes with a visible nucleus (upper left, the nucleus is indicated by the white dotted circle; bottom left, the oocyte does not have a nucleus). Scale bars: 20 µm. (ii) Scores of nucleus detection (p) on the test dataset (recall=TP/(TP+FN); precision=TP/(TP+FP), where TP=true positive, FN=false negative, FP=false positive) of ten neural networks (with random weights initialization) after training. Boxes represent the 25–75th percentiles, whiskers show the data within 1.5× from the interquartile range and the median is marked with a line. (D) Automatic determination of the NEBD timing in mouse oocyte maturation movies. (i) Nucleus detection score (p) in individual images of one movie, acquired at 10 min, 25 min, 1 h, 8.5 h and 12 h after movie beginning, calculated with the trained neural network. Scale bar: 20 µm. (ii) Evolution of the nucleus detection score in time and automatic determination of NEBD timing as the first transition from low score (before NEBD) to high score (after NEBD). (iii) Comparison of automatically and manually determined NEBD timing on 46 test oocyte maturation movies. *R*-square of the timing differences is indicated. The dashed line represents the *y*=*x* line. (iv) Histogram of the timing of NEBD from 424 movies. The dashed vertical line represents the 0.95 quantile above which oocytes are considered to present a delay in NEBD timing.

At the end of its growth in the ovary, an oocyte follows a crucial phase called meiotic maturation, which determines its capacity to be fertilized and sustain early embryonic development. Meiotic maturation consists of two successive highly asymmetric divisions in size (Mogessie et al., 2018; Verlhac and Terret, 2016). The first meiotic division (meiosis I) begins for an oocyte initially in prophase I with the rupture of the nuclear envelope (nuclear envelope breakdown, NEBD, Fig. 1A) and finishes with the formation of two daughter cells: a large oocyte and a small polar body (PB) (PB extrusion, Fig. 1A). This highly asymmetric division allows the oocyte to retain most of its cytoplasmic content accumulated during its growth, which is essential for early embryonic development (Mogessie et al., 2018; Verlhac and Terret, 2016; Coticchio et al., 2015). The oocyte then enters the second meiotic division (meiosis II) and arrests in metaphase II until fertilization by the sperm. This maturation step is particularly error-prone in terms of chromosome segregation and responsible for most aneuploidies in human (Mihajlović and FitzHarris, 2018; Nagaoka et al., 2012). Strikingly, in humans, the quality of oocytes decreases with maternal age, which constitutes a major societal issue in modern societies in which women tend to postpone childbearing. Oocyte maturation is thus the object of intense research efforts both in clinics and in academia, which often relies on using surrogate models similar to humans to analyze larger cohorts of genetically modified oocytes.

In the context of assisted reproductive technologies, *in vitro* oocyte maturation can be used to avoid hormonal treatments for women experiencing repeated *in vitro* fertilization failures or those who do not respond to hormonal stimulation. Moreover, oocytes can also be extracted and frozen for fertility preservation (Hatırnaz et al., 2018; Segovia et al., 2017). The success rate of *in vitro* oocyte maturation is typically around 70% (Kim et al., 2004), but the developmental potential of *in vitro*-matured oocytes is much lower than that for *in vivo*-matured ones (Kim et al., 2004; La et al., 2019; Monti et al., 2017). To improve *in vitro* oocyte maturation protocols, we need to better decipher the parameters associated with successful oocyte development. Several characteristics correlating with oocyte quality have been identified, such as the composition and amount of accumulated maternal mRNAs, the sizes of the oocyte, its zona pellucida (ZP) and its polar body, its cytoplasmic organization, or the presence of certain epigenetic modifications (Cran, 1985; Eppig et al., 1994; Fair et al., 1995; Hyttel et al., 1989; La et al., 2019). However, a consensus on the individual power of these parameters to predict the developmental potential of oocytes has never been reached, and there are often conflicting results (Bartolacci et al., 2022; Rienzi et al., 2011). Machine learning techniques can be used to tackle this problem as they can automatically identify more relevant features and take into account multiple features at the same time. These techniques have been applied to predict metaphase II oocyte or blastocyst quality and select the best embryo for implantation (Khosravi et al., 2019; Wang et al., 2019; Cavalera et al., 2019; Manna et al., 2013), but not to predict the potential of prophase I oocyte maturation (Fernandez et al., 2020; Zaninovic and Rosenwaks, 2020).

In both academia and clinics, computational tools to better characterize the developmental potential of oocytes are thus necessary. To follow live oocyte development, fluorescence imaging is commonly used in academia. However, it is limited to the number of fluorescent markers that can be visualized in parallel and it produces phototoxicity, potentially affecting the proper development of oocytes (Kasprowicz et al., 2017; Ounkomol et al., 2018; Skylaki et al., 2016; Vicar et al., 2019). Label-free imaging is a promising non-invasive method to overcome these hurdles (Kasprowicz et al., 2017) and is exclusively used in clinics. However, label-free images present a much lower contrast, making the segmentation of the object contour a limiting step (Buggenthin et al., 2013; Tse et al., 2009). Deep learning approaches provide a good performance for segmentation of label-free images from various cell types (Falk et al., 2019; Kim et al., 2019; Ronneberger et al., 2015). Therefore, we chose a versatile approach to implement a segmentation tool that would be robust to the diversity of oocyte types and modes of acquisition (mouse oocytes coming from academia and human oocytes from clinics), as well as to external objects present in the field of view, such as follicular cells or injection pipettes.

Here, we developed a machine learning approach to characterize and predict oocyte maturation *in vitro* based on images acquired non-invasively. First, we propose a new Fiji plugin, Oocytor, available open-source on GitHub (see Materials and Methods). Oocytor is a user-friendly tool to segment the contour of oocytes from diverse species as well as their zona pellucida, when present, from transmitted light images. Oocytor was designed to extract numerous morphological features and to automatically detect NEBD, which marks the beginning of the maturation process. Next, we implemented a machine learning approach to characterize and predict oocyte development. We decided to favor interpretability over predictive power and chose an approach based on hand-engineered features extracted from our plugin. We designed a list of 118 morphological features identified as potential markers of oocyte quality (Bartolacci et al., 2022; Ozturk, 2020; Rienzi et al., 2011). We showed that this machine learning approach can be used to discriminate oocyte populations and automatically identify morphological differences coming from a mutant strain. This automatic phenotyping could be a valuable tool for fundamental research studies. Finally, we used our pipeline to predict the potential of oocytes to start and perform a correct maturation. In addition to predicting oocyte quality before its maturation, it allowed us to identify the most determinant morphological features controlling the maturation process. As a proof of concept, we based our study on the *in vitro* maturation of mouse oocytes, which are relatively similar to human oocytes (Anderiesz et al., 2000; Levi et al., 2013; Ménézo and Hérubel, 2002), and offering access to more data and possible genetic manipulations. We tested how the results obtained on mouse oocytes could be transferred to *in vitro* human oocytes with a small dataset acquired from clinics.

## RESULTS

To maximally avoid perturbations and to be in line with clinical practice, our method to analyze oocyte development was based on non-invasive measurements. Therefore, we propose here a computational tool to characterize oocytes from images or movies acquired in transmitted light, without any fluorescent markers. Our first objective was to build a user-friendly computational tool to extract quantitative information from images and/or movies of oocytes undergoing maturation. For this, we acquired 468 movies of mouse oocytes, starting shortly before NEBD until metaphase II onset (Fig. 1A). Note that we made these movies freely accessible on Zenodo (see Materials and Methods).

### Overview of our new machine learning pipeline to characterize oocytes

To automatically analyze oocyte maturation, we propose a machine learning pipeline (Fig. 1B, overview of the pipeline and its different steps) based on our image dataset. First, movies or images were preprocessed for homogenization (see Materials and Methods). Then, the time of maturation initiation, characterized by NEBD, was automatically determined and used as a temporal landmark (Fig. 1B, Temporal alignment step). Next, images were segmented to identify the oocyte and its zona pellucida contours (Fig. 1B, Segmentation step), allowing the extraction of a vector of 118 numerical features describing an oocyte (Fig. 1B, Features extraction step). We then used these features, or an uncorrelated subset of them, in several machine learning methods according to our needs (Fig. 1B, Analysis step). We implemented the Temporal alignment, Segmentation and Features extraction steps into a Fiji plugin, Oocytor, proposing a user-friendly source to extract quantitative information from oocytes in transmitted light. Below we present more details on these three main steps of our pipeline.

### Automatic detection of NEBD from mouse oocytes

Oocyte maturation is a precisely, temporally regulated process. The time after NEBD is commonly used as a landmark to describe progression into oocyte maturation. When maturation is triggered *in vitro*, the oocyte population does not undergo NEBD perfectly synchronously, with few oocytes being extremely delayed and some remaining arrested in prophase I. We therefore designed an automatic assessment of NEBD in our plugin to annotate this event and temporally align time-lapse movies of oocyte development.

We first trained a neural network to determine the presence or absence of a visible nucleus. Our network takes as input an image containing one oocyte and evaluates the probability that it has already undergone NEBD (Fig. 1C, left panel, nucleus highlighted in dotted white circle). The architecture of a neural network determines its performance for a given task. We chose an architecture based on the neural network VGG-16 (Simonyan and Zisserman, 2015 preprint), which is specialized for image classification (Fig. S1A). We generated a dataset of mouse oocyte images manually annotated as before or after NEBD from our dataset (see Materials and Methods). Our final database comprised 7713 images, 90% of which were used for selection and training of the network (Fig. S1B, see Materials and Methods) and 10% for testing its performance. We measured network precision and recall (Taha and Hanbury, 2015), which give an indication of the quality and quantity of ‘hits’ (here, oocytes having undergone NEBD). We obtained a higher precision than recall (median score of 98% versus 96%, Fig. 1C, right panel), indicating that the network had a slight tendency to generate false negatives, i.e. oocytes without a nucleus that were predicted as still containing one. These false negatives might be due to the presence of a visible polar body in some images, which somehow confused the network. Indeed, the fourth image in Fig. 1D with a visible polar body has a lower score compared to the third and fifth images without a visible polar body.

We corrected this problem by considering the overall information in the movie instead of looking only at a single snapshot, and implemented NEBD determination in Oocytor. Each image from a movie was run through our trained neural network, which calculated a probability of the oocyte state being after NEBD for each time point (Fig. 1D, top panel). NEBD was set as the first transition point at which the nucleus detection score switches from low to high (Fig. 1D, bottom left panel). To evaluate the accuracy of our method, we compared the NEBD timings obtained with our plugin to the annotated ones from test movies, which were not used for training the network. Overall, automatically detected and manually assigned timings were very close (Fig. 1D, bottom middle panel), which confirmed that our plugin can reliably be used for automatic detection of NEBD. Finally, we calculated the NEBD timing for our 468 mouse oocyte maturation movies. We manually checked the movies for which the obtained NEBD time was delayed. We found that eight of the 44 oocytes that did not undergo NEBD were erroneously labeled as having a late NEBD and thus manually corrected. In the majority of the cases, NEBD occurred in oocytes (424 cells) approximately 30 min after the start of the recordings (Fig. 1D, bottom right panel). However, some oocytes required more time to undergo NEBD, which might reflect states of unfinished growth or other defects. Taking the delay in NEBD timing into account is important as it can reflect inherent differences in oocyte potential and is also essential to compare oocytes at the same stage of meiotic maturation. Oocytor allows us to do this automatically and to avoid manual annotation, which could be tedious for large datasets.

### A robust and generic segmentation pipeline of oocyte contours

**Fig. 2.**
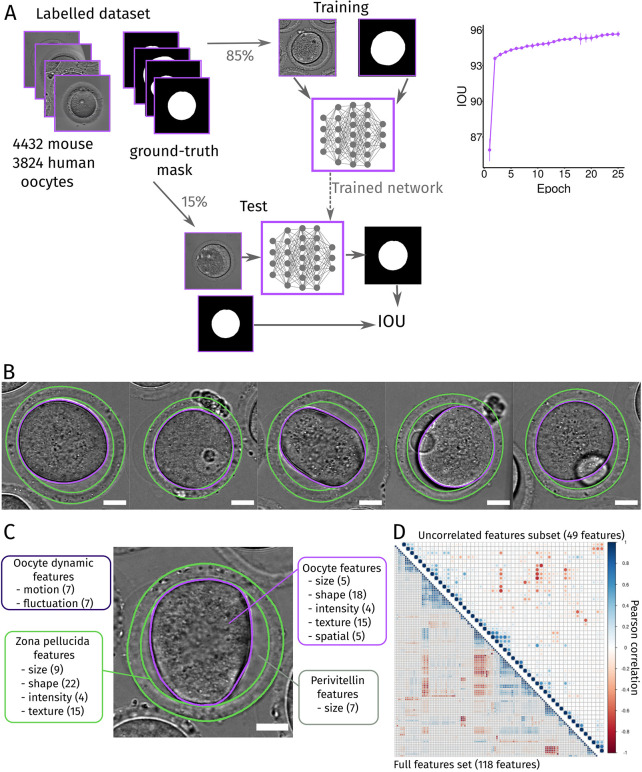
**Segmentation and extraction of features from oocytes.** (A) Scheme of the process used to segment the oocyte contour with neural network. Thousands of mouse and human oocyte images acquired under different conditions (top left panel) with their associated ground-truth (true segmentation mask, top left panel) were split into a training dataset (85% of the images, top middle panel) and a test dataset (bottom panel). Network score is evaluated by the intersection over union (IOU) score during the training iterations (epochs) of the network (top right graph). Once trained, the performance of the network is evaluated by measuring the IOU between its output and the ground truth on the test dataset (bottom panel). (B) Examples of segmentation of the oocyte membrane (purple lines) and of the zona pellucida contours (green lines) obtained with Oocytor on mouse oocytes at different stages of maturation. Scale bar: 20 µm. (C) Features characterizing an oocyte. The numbers of features are shown in parenthesis and the features are grouped by categories: describing the oocyte (purple), its zona pellucida (green), the perivitelline space in between (gray) and the dynamics of the oocyte (dark purple). Scale bar: 20 µm. (D) Features correlation and subset selection. Pearson correlation coefficient calculated for each pair of the 118 features (bottom graph) from the values obtained from the training on mouse oocytes dataset. After subset selection (absolute Pearson coefficient under 0.75), 49 uncorrelated features were kept (top graph) and used in machine learning algorithms.

The second step of our machine learning pipeline was to extract quantitative and interpretable information from images to better describe oocytes. For this, we first implemented a deep learning-based tool to segment the contour of the oocyte from images acquired in transmitted light. We based our neural network (Fig. S1C) on the U-Net architecture (Ronneberger et al., 2015), specifically designed for segmentation of biomedical images (Caicedo et al., 2019; Saleh et al., 2019). First, we created a database of thousands of images of single oocytes with their corresponding segmentation, which are our ground-truth labels. With the aim of building a robust and versatile network (Möckl et al., 2020), we pooled together datasets of both mouse and human oocytes, derived from different contexts (see Materials and Methods). We obtained a database of 8256 images of which 85% were used for selection and training of the network and the remaining 15% for testing it (Fig. 2A). During training, the network learned by optimizing its parameters to minimize the error between its outputs and the ground-truth images (Fig. 2A, right panel). We evaluated this error by the intersection over union (IOU) score, a metric measuring segmentation quality (Taha and Hanbury, 2015). The implementation of the network requires choosing additional parameters (e.g. the number of iterations) called hyper-parameters. Their fine tuning was performed on the training dataset with a cross-validation technique (see Materials and Methods; Fig. S1D). We then trained the selected network (Fig. 2A, left). For mouse oocytes, we obtained an average IOU of 97% on the test dataset (Fig. S1F). We applied the same steps to select and train neural networks to determine the contours (inner and outer limits) of the zona pellucida, a glycoprotein layer surrounding the oocyte, for which we built a database of 3578 images (see Materials and Methods). We obtained an average IOU of 82% for the zona pellucida segmentation on the mouse test dataset (Fig. S1E,F). Eventually, we implemented this step in our Fiji plugin with the objective of proposing a user-friendly tool for segmentation of oocyte and zona pellucida contours from transmitted light images to the oocyte research community (see Materials and Methods; Fig. S1G). Fig. 2B shows examples of segmentation obtained with Oocytor for oocytes at different stages of maturation. Oocytor can thus be used to segment oocytes from transmitted light images and, based on how it was constructed, should be relatively robust to different imaging conditions.

### A numerical description of oocytes

To numerically characterize oocytes at a given time point, we defined 118 quantitative measures of their properties (features). These features described the oocyte, its surrounding zona pellucida, its perivitelline space (space between the oocyte and its zona pellucida) and its dynamics (Fig. 2C). For this, we used radiomics features (Fornacon-Wood et al., 2020; Rizzo et al., 2018), morphological characteristics that have been shown to be important to discriminate oocyte quality (Inoue et al., 2007; Ozturk, 2020; Rienzi et al., 2011), and other characteristics that could be applied to the oocyte description. We implemented the measures of all features based on the segmentation of oocyte and zona pellucida contours from images acquired in transmitted light using our plugin (see Materials and Methods). We defined a high number of features in order to have an unbiased exploration of the oocyte. It is important to note that some features can measure related biological properties and thus have correlated values (Fig. 2D, bottom graph). Several methods are available to extract a subset of independent features necessary for some machine learning algorithms (Hira and Gillies, 2015). Here, we implemented an unsupervised subset selection based on correlation (see Materials and Methods; Fig. 2D, top graph). Our 118 features or the uncorrelated subset can then be used in machine learning algorithms to classify and characterize oocyte populations.

### A powerful pipeline to automatically phenotype oocytes

**Fig. 3.**
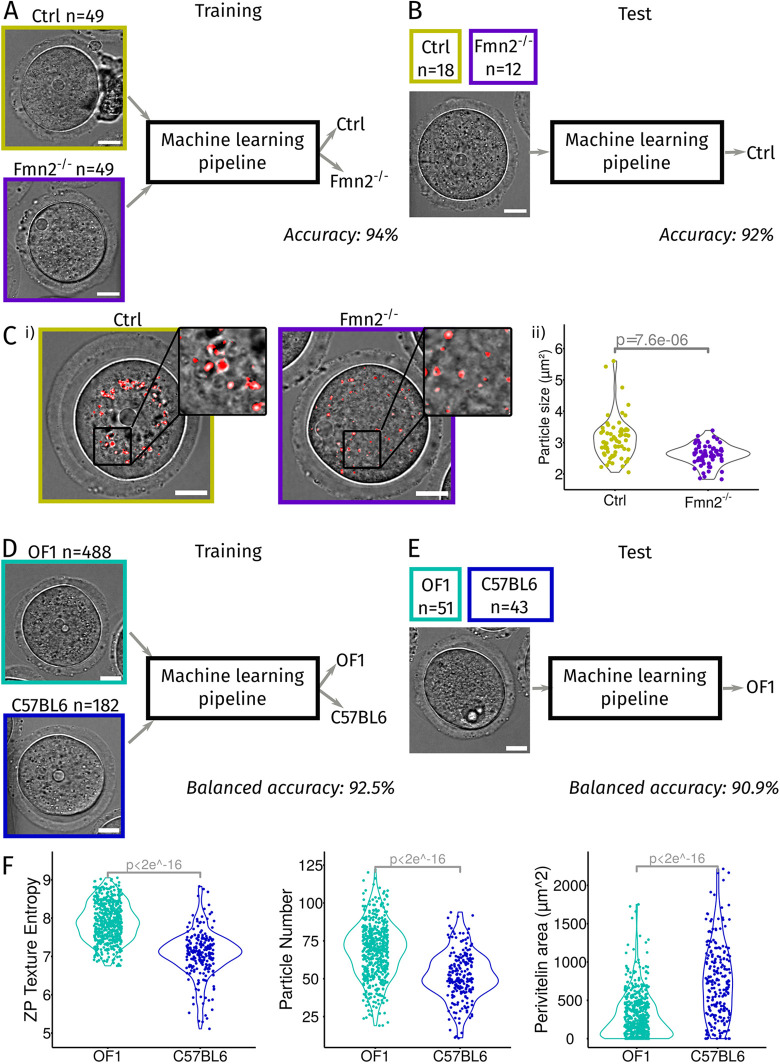
**Oocyte phenotyping using our machine learning pipeline.** (A) Training of our machine learning pipeline to recognize control (Ctrl; WT or *Fmn2^+/−^*) and Formin-2-knockout (*Fmn2^−/−^*) oocytes. A training database of 49 Ctrl (dark yellow) and 49 *Fmn2^−/−^* (dark purple) oocytes was built from two independent datasets and used to train our machine learning pipeline to discriminate Ctrl and *Fmn2^−/−^* oocytes. The accuracy of the classification of oocytes as Ctrl or *Fmn2^−/−^* was evaluated around 94% (cross-validation on the training data). Scale bars: 20 µm. (B) Test of our machine learning pipeline with the selected random forest algorithm. A new dataset of 18 Ctrl (dark yellow) and 12 *Fmn2^−/−^* (dark purple) oocytes was run through the pipeline and the accuracy of the classification of oocytes as Ctrl or *Fmn2^−/−^* was evaluated to be around 92%. Scale bar: 20 µm. (C) Cytoplasmic particle size is the second most discriminant feature between Ctrl and *Fmn2^−/−^* oocytes. (i) Example of particles detected and measured in our plugin (red dots) in Ctrl (dark yellow box) and *Fmn2^−/−^* (dark purple box) oocytes (scale bars: 20 µm). Insets correspond to a zoom of the image for better visualization. (ii) Comparison of the mean cytoplasmic particle size from Ctrl and *Fmn2^−/−^* oocytes. Statistical comparison was assessed using a Kolmogorov–Smirnov test (*P*-value indicated on the graph). (D) Training of our machine learning pipeline to discriminate oocytes coming from two wild-type mouse strains: OF1 and C57BL6. A training database of 488 OF1 (light blue) and 182 C57BL6 (dark blue) oocytes was built from several independent datasets (∼20 experiments from three projects) and used to train our machine learning pipeline. The balanced accuracy of the classification of oocytes was evaluated around 92.5% (fivefold cross-validation on the training data). Scale bar: 20 µm. (E) Test of our machine learning pipeline with the random forest algorithm. A new dataset of 51 OF1 (light blue) and 43 C57BL6 (dark blue) oocytes was run through the pipeline and the balanced accuracy of the classification of oocyte was evaluated around 91%. Scale bar: 20 µm. (F) Most discriminant features between the population of oocytes coming from the two wild-type mouse strains. Comparison of the values for OF1 (light blue) and C57BL6 (dark blue) oocytes are shown. *P*-values indicated on the graphs were calculated with a Kolmogorov–Smirnov test. From left to right panels: graphs show the comparison of the average value of the entropy of the texture of the zona pellucida (measured by Haralick's entropy), the average number of detected cytoplasmic particles and average perivitelline area.

In fundamental research, genetic manipulation is often performed to study a particular aspect of oocyte maturation. Our machine learning pipeline allowed us to build an algorithm that, after training, should automatically recognize the population of origin of an oocyte. The success or failure of this classification allows us to test whether the population presents morphological differences and oocytes could be sorted according to the extent of their mutant phenotype. To demonstrate the potential of our pipeline, we chose to test it on a well-characterized population of Formin-2-knockout oocytes (*Fmn2^−/−^*) against control mouse oocytes [wild type (WT) and *Fmn2^+/−^*], for which we already have datasets of movies of oocytes arrested in prophase I (Al Jord et al., 2021 preprint; Almonacid et al., 2015). We kept the smaller dataset to test the performance of our approach and trained the algorithm with the other two combined datasets (Fig. 3A). We first verified visually that our segmentation with Oocytor performed well on these new data, despite the differences in oocytes phenotypes and modes of image acquisition (Fig. S2A). We then applied our pipeline to classify oocytes by their population of origin. For this, in the Analysis step of the pipeline, we compared the performance of different machine learning algorithms (see Materials and Methods; Fig. S3B) that received the features describing one oocyte as input and classified it either as control (Ctrl) or *Fmn2^−/−^* (Fig. 3A). We obtained the best performance with the random forest algorithm (Breiman, 2001), which was able to recognize control and *Fmn2^−/−^* oocytes with approximately 94% accuracy on the training data (Fig. 3A; Fig. S2B,C). We tested the performance of this algorithm on an independent set of data and obtained a classification accuracy of about 92% (Fig. 3B), with a better recall than precision (Fig. S2C). Hence, even with a small number of training oocytes (49 control and 49 *Fmn2^−/−^* oocytes), our algorithm was able to discriminate oocytes based on their phenotype, owing to their morphological description.

To understand how the algorithm recognized the two oocyte populations, we examined the weight of the different features (based on the Gini index, see Materials and Methods). The average cytoplasmic agitation, measured by particle image velocimetry (Tseng et al., 2012), was the most discriminant feature for the algorithm (Fig. S2D,E). Indeed, cytoplasmic agitation was strongly reduced in *Fmn2^−/−^* oocytes (Fig. S3E), confirming previous work showing that cytoplasmic agitation is controlled by the movement of actin-coated vesicles nucleated by Formin-2 (Almonacid et al., 2015; Holubcová et al., 2013). In addition, the second and third most discriminant features were the size and spatial distribution of the cytoplasmic particles (Fig. S2D). The difference in spatial repartition most likely reflects the difference in the position of large objects such as the nucleus (centered in control oocytes, off-centered in *Fmn2^−/−^* oocytes, Fig. 3A; Almonacid et al., 2015). The difference in the average particle size (bigger in control oocytes, Fig. 3C), a newly identified feature, could be explained by the difference in cytoplasmic agitation, which tends to center large objects in the cytoplasm (Colin et al., 2020) and thus could favor the aggregation of particles into clusters.

To further challenge our pipeline phenotyping capacities, we applied it to discriminate oocytes with more subtle differences, derived from two different mouse wild-type strains. Indeed, we had access to single images of oocytes taken before NEBD from different strain backgrounds, OF1 and C57BL6 (see Materials and Methods). We trained our pipeline to recognize the strain of origin (Fig. 3D) and tested its performance on a dataset not used in the training (Fig. 3E; Fig. S2F). The pipeline was able to classify oocytes correctly with a balanced accuracy around 91%, with a better precision than recall (Fig. S2F). Moreover, our pipeline indicated that the most discriminant features (based on the Gini index) to discriminate the strain of origin were the texture of the zona pellucida, the texture of the cytoplasm, the number of cytoplasmic particles and the size of the perivitelline space (Fig. S2G; Fig. 3F). Thus, although they were control oocytes, the two strain backgrounds presented morphological differences that were revealed by our algorithm, which showed the importance of using the same genetic background in experiments or to be aware of the strain differences.

These results validate and highlight the power of our approach to automatically identify the main differences between two oocyte populations (wild-type or mutant backgrounds) and discover novel characteristics (i.e. particle size for Formin-2-knockout oocytes) associated with a mutant background. We believe that this approach for oocyte phenotyping might be of particular interest in basic research.

### Our pipeline predicts mouse oocyte maturation outcome before entry into meiosis

**Fig. 4.**
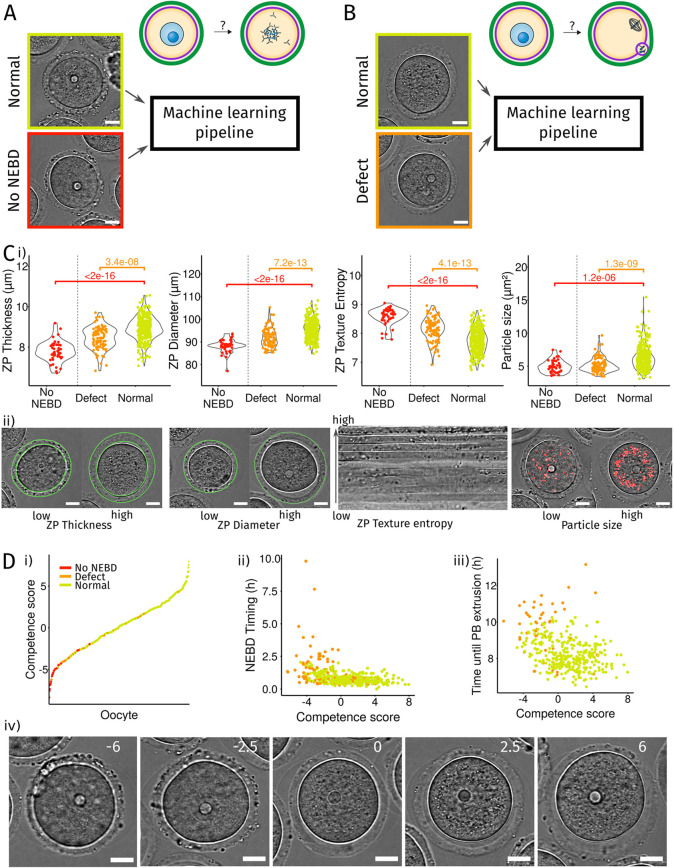
**Prediction and characterization of mouse oocyte maturation with our machine learning pipeline.** (A) Prediction of failure to enter meiosis I. Our machine learning pipeline is trained with our mouse oocyte maturation dataset. Each oocyte was labeled as ‘Normal’ if it underwent NEBD (yellow box, 424 oocytes) and ‘No NEBD’ otherwise (red box, 44 oocytes). Scale bars: 20 µm. (B) Prediction of a defect in maturation after meiosis resumption. Each oocyte was labeled as ‘Normal’ if no defect was detected (yellow box, 327 oocytes) and as ‘Defect’ if the oocyte did not extrude a first polar body, resorbed its polar body, entered meiosis I after an abnormal delay or extruded its polar body after a delay (orange box, 96 oocytes). Scale bar: 20 µm. (C) Discriminant features for NEBD failure or maturation defect. (i) Comparison of the values of the discriminant features for oocytes that do not enter meiosis I (red, No NEBD), with maturation defect (orange, Defect) or normal (yellow, Normal). *P*-values indicated on the graphs were calculated with a Kolmogorov–Smirnov test. From left to right: graphs show the comparison of the average thickness of the zona pellucida (average distance between the inner and the outer contours), average value of the zona pellucida outer diameter, average value of the entropy of the texture of the zona pellucida (measured by Haralick's entropy) and average cytoplasmic particle size. (ii) Illustration of oocytes with low and high values of the discriminant features. The ZP contours are highlighted in green, cytoplasmic particles in red. Scale bars: 20 µm. (D) Quantitative measure of oocyte competency. The competence score is defined as a linear combination of the four features presented in C: competence score=ZP outer perimeter−ZP texture entropy+ZP thickness+Cytoplasmic particle size. (i) Sorted competence scores from all oocytes of the mouse maturation dataset. Each point represents one oocyte, and the color of the category to which the oocyte belongs: no NEBD (red), maturation defect (orange), normal (yellow). (ii) Timing of NEBD according to the oocyte competence score. (iii) Time spent between NEBD and first polar body extrusion according to the oocyte competence score. (iv) Examples of oocytes observed in transmitted light presenting different competence scores indicated in white on the images (bottom panel), increasing from left (very low competence score) to right (high competence score). Scale bars: 20 µm.

Another major use of our pipeline is its predictive capacity. We used it to predict meiotic entry and the maturation potential of an oocyte arrested in prophase I before its division. Out of the 468 movies from our dataset of mouse oocyte maturation, 44 oocytes never resume meiosis. We first tested whether we could predict this developmental failure before it happens (Fig. 4A). For this, we trained our machine learning pipeline to classify which oocytes would undergo NEBD or not, based on the average value of features over the first 12 min of the movies. By cross-validation, we selected the random forest algorithm that performed the best for this task (see Materials and Methods; Fig. S3A). Finally, we trained our selected algorithm on all the data and tested its performance on a new independent dataset of 69 oocytes, seven of which did not resume meiosis. As the dataset was strongly imbalanced between oocytes that would resume meiosis or not, we measured the balanced accuracy of the pipeline prediction (see Materials and Methods) and obtained a balanced accuracy of about 91% (Fig. S3C). Therefore, our algorithm has the power to correctly predict which prophase I oocytes will fail to undergo NEBD. The most discriminant features associated with meiotic entry were the thickness and texture of the zona pellucida and the size of the oocyte–zona pellucida complex (Fig. S3B). Oocytes that remained arrested in prophase I were smaller, with a thin and heterogeneous zona pellucida (Fig. 4C, red), consistent with them not completing their growth (Wassarman and Litscher, 2012), further validating our approach.

Next, we aimed to predict and characterize the potential of oocytes to properly mature (Fig. 4B). We analyzed only the movies of oocytes that underwent NEBD and temporally aligned them based on the NEBD timing calculated with Oocytor. We considered that oocytes had maturation defects when they did not extrude or when they resorbed their first polar body (PB), or if the timing of NEBD or first polar body extrusion was abnormally late (Fig. S4). We trained machine learning classifiers to predict oocytes that will have a maturation defect based on the average values (over 12 min) of the features taken 15 min before NEBD. Our selected algorithm (balanced random forest, Fig. S3F) had a balanced accuracy of nearly 80% on the training data (Fig. S3D) and 90% on the test dataset (which contained three out of 62 oocytes with maturation defects, Fig. S3F). Thus, our approach allowed the prediction of the maturation potential of oocytes before maturation even resumed.

We then compared the performance of our pipeline by comparing the accuracy of the predictions made with that of predictions made by a small pool of scientists, using images of oocytes 15 min before NEBD. The performance of our pipeline in predicting NEBD success was in a range similar to the predictions made by scientists with a strong expertise in oocyte maturation (Fig. S3C). Both sets of predictions were much more accurate than the ones from novice scientists (Fig. S3C), showing the necessity of training for both human and machine predictions. In all predictions (pipeline or humans), the precision was better than the recall (Fig. S3C): oocytes predicted to resume meiosis did indeed resume meiosis whereas some oocytes that showed a potential to resume meiosis were not identified as such. Importantly, the pipeline predictions outperformed human expert ones for the prediction of maturation defects (Fig. S3F). Thus, our pipeline can be used as a tool to automatically screen large numbers of oocytes to predict NEBD or maturation defects, without the need of human expertise. In addition, by providing better predictions than those from human experts for an event occurring after a period of *in vitro* culture lasting a minimum of 10 h, our pipeline can save time and resources by pointing out bad oocytes early on.

### Oocyte quality can be scored from four non-dynamic features

The two most discriminant features used by the algorithm for the prediction of oocyte maturation outcome were the zona pellucida texture and the cytoplasmic particle size (Fig. S3E). Zona pellucida texture (measured by Haralick's entropy) was a determinant feature of oocyte quality for both the entry and the success of oocyte maturation, making it highly relevant for assessing oocyte potential. This feature reflects the heterogeneity of the zona pellucida (Fig. 4C, third panel). It was highest in oocytes that did not start maturation (Fig. 4C, third panel, red) and was relatively high in oocytes that had maturation defects (Fig. 4C, third panel, orange). The second discriminant feature for successful oocyte maturation was cytoplasmic particle size, which was smaller in oocytes that failed to resume or to properly end meiotic maturation (Fig. 4C, fourth panel, red and orange). Finally, both the thickness of the zona pellucida and the size of the oocyte–zona pellucida complex were good indicators of oocyte quality (Fig. 4C, first and second panels). Thus, the characteristics of the zona pellucida are a good measure of oocyte maturation potential.

To evaluate the quality of an oocyte, we defined a score based on these most relevant features for resuming and completing maturation. This competence score, as an indicator of oocyte quality, which corresponds to its capacity to develop properly, was defined as a linear combination of the standardized values of the four most discriminant features: texture and thickness of the zona pellucida, size of cytoplasmic particles and perimeter of the outer limit of the zona pellucida (Fig. 4D; see Materials and Methods). Importantly, this score was based on non-dynamic features and, therefore, only required a single image acquired from an oocyte arrested in prophase I. Consistently, most oocytes that did not resume meiosis had a low competence score (Fig. 4D, left panel, red), whereas oocytes that matured properly had an overall high competence score (Fig. 4D, left panel, yellow). Moreover, the competence score correlated with the maturation dynamics of oocytes that entered meiosis I: oocytes with a low score resumed meiosis more slowly (Fig. 4D, middle panel, Pearson coefficient *r*=−0.4, *P*<10^−16^) and were delayed in the first PBE extrusion (Fig. 4D, right panel, Pearson coefficient *r*=−0.36, *P* =4.8×10^−13^). Hence, our competence score allows the ranking of oocytes arrested in prophase I by their developmental potential (Fig. 4D, bottom panel).

### Heterogeneity of the zona pellucida is predictive of *in vitro* maturation of human oocytes

As our algorithm proved to accurately estimate the quality of mouse oocytes, we then tested whether the same discriminating features would be applicable to human oocytes. For this, we used a dataset of 72 human oocyte maturation movies acquired in clinics (see Materials and Methods). Oocytes were collected from patients after hormonal stimulation and those that did not complete maturation, which were therefore unsuitable for intracytoplasmic sperm injection (ICSI), were followed overnight for research purposes. The dataset was a mix of prophase I and meiosis I oocytes at the beginning of the movies, hence it could not be temporally aligned as for mouse oocytes, increasing the variability. Out of 72 oocytes, 48 oocytes eventually succeeded in extruding a first polar body. To test whether our previously identified features could predict polar body extrusion, we used our plugin on these movies. As we trained our neural networks for cortex and zona pellucida segmentation on both mouse and human oocytes, we achieved a good performance on the segmentation of human oocytes, despite the presence of obstacles in the field of view, such as the presence of cumulus cells (Fig. S5A). This demonstrates that Oocytor can successfully segment human oocyte and zona pellucida contours. Using Oocytor, the values of the 118 morphological features were then extracted. To visualize the distribution of the values of these features at the beginning of the movies, the uniform manifold approximation and projection (U-MAP) reduction technique (McInnes et al., 2018 preprint) was applied. No strong morphological differences were detected between extruding and non-extruding oocytes (Fig. S5B, left panel). We then compared extruding and non-extruding human oocytes using the values of the discriminant features identified from mouse oocytes at the beginning of the movies (Fig. S5B). The differences were not significant, except for the texture of the zona pellucida (Fig. S5B, middle and right panels). As those movies could not be temporally aligned, the analysis was performed on the values of the features at the beginning of the movies; hence, the oocytes were at different stages of development, contrary to our analysis on mouse oocytes, which increased the variability of the values of the features. Moreover, it is important to remember here that the population of human oocytes analyzed in these experiments corresponded to oocytes not responding to the hormonal stimuli and, thus, were probably of lower quality. Hence, this population might not be comparable to the mouse population and might not represent a physiologically relevant cohort of human maturing oocytes. Consistent with this, nuclei, which were visible at the beginning of 39 movies, were generally not centered, a feature known to correlate with defects in polar body extrusion (Levi et al., 2013).

Nevertheless, this analysis of low-quality human oocytes before metaphase II, the only ones accessible so far for research purposes, showed the importance of the texture of the zona pellucida: a more heterogeneous zona pellucida correlated with maturation defects in both human and mouse oocytes. This feature has also been reported as an important marker of the quality of human matured oocytes and human embryos, to assess their potential to develop to the blastocyst stage and their implantation success rate (Rienzi et al., 2011). In particular, it was shown that the optical birefringence measured by polarized light microscopy was higher and more uniform in human oocytes that developed properly (Montag and van der Ven, 2008). Altogether, our results suggest a crucial role of the zona pellucida in oocyte maturation and reveal that its morphology can reflect the quality of the oocyte even before maturation.

### Generalization of Oocytor to oocytes coming from other species

**Fig. 5.**
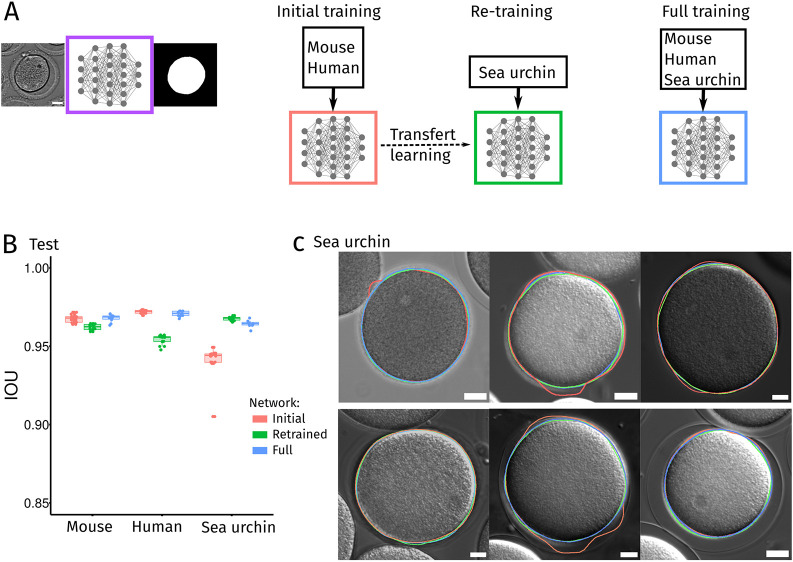
**A generic pipeline to segment oocytes from different species.** (A) Segmentation of sea urchin egg membrane using Oocytor. Eggs were segmented using our neural network trained on mouse and human oocytes presented in this paper (Initial, light red). A new neural network was trained specifically on sea urchin eggs, starting from our initial neural network with transfer learning (Retrained, green). Finally, a complete neural network was trained from scratch on all the oocytes species: mouse, human, sea urchin (Full, blue). (B) Performance of the segmentation with the three types of neural networks training: Initial (light red), Retrained (green) and Full (blue). The performance is measured as the intersection over union (IOU) score compared to a manually generated ground-truth, on our three test datasets of mouse, human and sea urchin oocytes. Boxes represent the 25–75th percentiles, whiskers show the data within 1.5× from the interquartile range and the median is marked with a line. (C) Examples of resulting segmentation of sea urchin eggs using our three neural networks: Initial (light red), Retrained (green) and Full (blue). Scale bars: 20 µm.

Here, we presented our open-source plugin Oocytor and showcased its use in a computational pipeline to screen mouse and human oocytes. We believe that our machine learning pipeline could easily be adapted to oocytes coming from other species as well. One of the pitfalls of machine learning algorithms and neural networks in particular is their major dependence on the data used to train them. In general, these networks perform very well on a dataset similar to those used for training, but can fail completely on new ones. We attempted to produce more robust and versatile networks by feeding them a variety of images (Möckl et al., 2020), pooling human and mouse oocyte images from different projects, acquired in different laboratories and clinics. Nonetheless, the networks could still yield limited results on oocytes never seen before by the networks, such as oocytes from a different species. In this case, a new network could be trained with only few images of these ‘foreign’ oocytes by using the transfer learning technique (Bengio, 2012) on our neural network. To test this, we used our neural network to segment the membrane of sea urchin eggs, a species not used in the training data. The resulting segmentation of sea urchin eggs was acceptable with an IOU score of about 94% (Fig. 5B,C, initial results in red), but not as good as those obtained on mouse and human datasets (IOU around 96–97%). As expected, using transfer learning (Fig. 5A) and retraining our neural network specifically on a few sea urchin eggs (*n*=185), we reached an IOU score of about 96.5% (Fig. 5B,C, retrained results in green). However, as this new network was retrained specifically on sea urchin eggs, its performance on mouse and human oocytes decreased (Fig. 5B,C, retrained results in green). Finally, we trained a neural network combining the three datasets: mouse, human and sea urchin oocytes (Fig. 5A). This strategy was successful and allowed us to propose a more general network performing as well on the three types of oocytes (Fig. 5B,C, final results in blue). This more general network is available on our GitHub repository and can be used as a basis to use Oocytor on other oocyte species. Moreover, the dataset used for training our neural networks (mouse, human and sea urchin oocytes) is freely accessible on Zenodo (see Materials and Methods), so that it can be used to train other neural networks.

## DISCUSSION

We developed a computational tool to segment and numerically describe oocytes, which was then associated with a machine learning pipeline to phenotype oocyte populations or predict and characterize oocyte meiotic resumption and maturation. Our tool was set up on images acquired in transmitted light, which allows its use both in academia and in clinics. As all the steps of the pipeline are automatized, our tool can be used to screen an important number of oocytes for large-scale studies.

To make our tool user friendly, we implemented it as a Fiji plugin (Schindelin et al., 2012), Oocytor, available open source on GitHub (see Materials and Methods). Oocytor can perform three tasks on label-free images: segmentation of the oocyte and its zona pellucida contours, NEBD detection and extraction of hundreds of morphological features. For the first two tasks, we took advantage of the power of deep learning algorithms and trained U-Net-like networks on mouse, human and sea urchin oocyte images that were pooled together for a better generalization (Fig. 5). Oocytor also offers the possibility to automatically define the NEBD timing of mouse oocytes, thus avoiding manual annotation and providing a temporal reference to align maturation movies. This property can turn out to be a true time-saver for studies implemented on large cohorts of oocytes. We implemented the measurements of 118 features, allowing the tool to numerically describe an oocyte at any stage of its development. This list could be further expanded. In particular, it would be interesting to add segmentation and features to better describe polar body properties, which certainly would help better characterization of metaphase II-arrested oocytes. New neural networks could also be trained to infer the position of the nucleus, spindle or other organelles from images acquired in transmitted light using data coupled with fluorescence labeling (Christiansen et al., 2018; Ounkomol et al., 2018; Wieslander et al., 2021). This approach is very promising for fundamental research to avoid fluorescence labeling. These steps of synchronization, segmentation and numerical description are independent and can then be used to answer a variety of fundamental research studies on oocytes.

Based on the numerical description of oocytes, we developed an interpretable machine learning pipeline to classify oocytes and identify major differences between oocyte populations. Here, we demonstrated that our algorithm can discriminate oocytes from different genetic backgrounds, such as control and *Fmn2^−/−^* oocytes, after training with only a small dataset and revealed major morphological differences between these two populations. This phenotyping could be very useful in the mouse oocyte research community, in which genetic modifications are often used as a tool to identify gene function without knowing all the consequences on oocyte characteristics. We thus propose a new approach to systematically identify new differences in an unbiased and automated manner and thus to potentially reveal novel gene function during oocyte development.

We then used this approach to explore oocyte quality within a wild-type population for clinical applications. We developed our approach on mouse oocytes, which allowed us access more data and possible manipulations. We successfully trained our pipeline to recognize mouse oocytes that failed to resume meiosis and to predict maturation defects before NEBD. Our pipeline performed similarly to human experts to predict meiosis resumption but outperformed human predictions for maturation defects. Oocytes that did not enter meiosis I were smaller with a thin and heterogeneous zona pellucida. It has been previously shown that small size is a sign of oocyte incompetence, which corresponds to oocytes unable to successfully develop to metaphase II (Hirao et al., 1993; Kanatsu-Shinohara et al., 2000; Wickramasinghe et al., 1991). This small size is most probably an indicator of unfinished oocyte growth. The zona pellucida thickness correlated with the size of the oocyte before NEBD in our mouse data (Pearson correlation *r*=0.42, *P*<10^−16^), consistent with the fact that the zona pellucida thickness and the oocyte diameter increase concomitantly during oocyte growth (Wassarman and Litscher, 2012). However, this correlation was lost after NEBD (Pearson correlation at NEBD+5 h *r*=0.18, *P*=1.6×10^−3^); the zona pellucida thickness remained constant, whereas the oocyte size decreased. Therefore, ZP thickness provides information on the maturation potential of the oocyte, which is different from the information provided by oocyte size. In human oocytes, no correlation has been found between zona pellucida thickness and oocyte diameter in metaphase II-arrested oocytes (Bertrand et al., 1995), consistent with our findings of low correlation in mouse oocytes. Moreover, in human embryos, zona pellucida thickness was positively associated with development to the blastocyst stage and embryo quality (Høst et al., 2002; Raju et al., 2007; Shen et al., 2005), but negatively correlated with fertilization success (Bertrand et al., 1995; Marco-Jiménez et al., 2012). Thus, zona pellucida thickness could be a relevant feature for assessing oocyte (our study) and embryo quality.

Our machine learning algorithm revealed that mouse oocytes with maturation defects had a more heterogeneous zona pellucida and smaller cytoplasmic particles (Fig. 4C). The heterogeneity of the zona pellucida, measured by Haralick's entropy, was a discriminant feature for both the initiation and the success of maturation. Moreover, this feature was also relevant for predicting human oocyte maturation potential. This texture could be related to the birefringence of the zona pellucida, which is used to assess human embryo quality (Gabrielsen et al., 2000; Ozturk, 2020; Raju et al., 2007; Rienzi et al., 2011) and reflects the structuration of the zona pellucida. Cytoplasmic particles were smaller not only in wild-type oocytes, with a low competence score, but also in *Fmn2^−/−^* oocytes (Fig. 3D); thus, this difference observed in the control oocyte population could be related to differences in cytoplasmic actin activity that was reduced both in oocytes of low competence scores and in *Fmn2^−/−^* oocytes (Fig. S4B, Fig. S2E). Previous studies showed that actin-coated vesicle activity follows a gradient from the cortex to the center, generating a non-specific centering force (Almonacid et al., 2015; Colin et al., 2020), which could favor cytoplasmic particle aggregation. This reduced activity, which correlated with a low maturation potential (Fig. S4B), was also associated with slower maturation dynamics (Fig. 4D, middle and right panels).

To conclude, our machine learning pipeline could predict oocyte maturation with a fidelity between 80% and 90%. Furthermore, we identified two new features, the texture of the zona pellucida and the size of cytoplasmic particles, as good predictors of the maturation potential of mouse oocytes. Moreover, we showed that the zona pellucida texture could be a good predictor of the maturation potential of human oocytes. We also defined a competence score to rank mouse oocytes by their capacity for maturation and revealed defects in the cytoplasmic activity in poor-quality oocytes. This ranking could be used to improve *in vitro* maturation protocols by identifying the most promising oocytes. By adding new features to Oocytor, such as polar body contour detection, it might also be possible to adapt our approach to assess the potential of oocytes for fertilization and embryonic development.

## MATERIALS AND METHODS

### Datasets

#### Mouse oocyte maturation

Our study was primarily based on a dataset of 468 movies of *in vitro* matured mouse oocytes. This dataset is publicly available on Zenodo (Letort et al., 2022a). An additional dataset of 69 mouse oocyte maturation movies were acquired independently, after the pipeline development, and used only to test its final performance.

OF1 or C57BL6J (referred to as C57BL6 in the main text) female mice (Charles River Laboratories) aged between 3 and 10 weeks were used for experiments. The prophase I-arrested oocytes were collected according to laboratory protocol in M2 medium made in-house with 4 g/l bovine serum albumin (BSA, A3311, Merck) supplemented with 1 µM milrinone (M4659, Merck) (Reis et al., 2006). For *in vitro* maturation, the oocytes were washed out from milrinone and cultured in M2 medium under mineral oil (M8410, Merck) at 37°C. They were then placed under a Leica DMI6000B microscope equipped with a Plan-APO 40×/1.25 NA oil immersion objective, a motorized scanning deck and an incubation chamber (37°C), a Retiga 3 CCD camera (QImaging, Burnaby) coupled to a Sutter filter wheel (Roper Scientific), and a Yokogawa CSU-X1-M1 spinning disk. Images were acquired using Metamorph (Universal Imaging, version 7.7.9.0) every 3 min in transmitted light with the objective 20×/0.75 NA for 20 h at 37°C.

All animal studies were performed in accordance with the guidelines of the European Community and were approved by the French Ministry of Agriculture (authorization no. 75-1170) and by the Direction Générale de la Recherche et de l’Innovation (DGRI; GMO agreement number DUO-5291).

#### *Fmn2^−/−^* dataset

We re-used datasets of control and *Fmn2^−/−^* genotypes of oocytes arrested in prophase I from previous studies [*Fmn2^+/−^*, *n*=25; *Fmn2^−/−^*, *n*=12 (Al Jord et al., 2021 preprint); WT, *n*=18; *Fmn2^−/−^*, *n*=12 (Almonacid et al., 2015)] and we performed a supplemental experiment with oocytes from both genotypes (WT, *n*=24; *Fmn2^−/−^*, *n*=37) to increase the training dataset (see protocol above). Oocytes were imaged every 1 min (movies were under-sampled for the dataset when the imaging frequency was higher) for 5 min or more. Datasets were not acquired with the same microscope objective resolution (0.1135 µm/pixel, 0.1613 µm/pixel and 0.227 µm/pixel), so we resized the images to a resolution of 0.1613 µm/pixel to allow direct comparison.

#### Human oocyte maturation

Human oocyte maturation movies were acquired at the Cochin hospital (Paris, France) for research purposes (72 movies). Immature human prophase I-stage and meiosis I-stage oocytes found in cohorts retrieved for the purpose of ICSI can be used for research according to the French legislation, with the consent from patients. The *in vitro* maturation study was performed with oocytes (*n*=72) that were donated for research by patients undergoing assisted reproductive technology protocols. It was approved by the Germetheque Biobank (BB-0033-00081) under the number 20160912. Once the ICSI procedure was performed for the patients, oocytes that did not reach metaphase II were included in the protocol. All clinical investigation was conducted according to the principles expressed in the Declaration of Helsinki. Patients were not renumerated. Culture dishes were prepared, covered with mineral oil (Irvine Scientific, Ireland), warmed and pre-gassed before *in vitro* maturation. Cumulus cells had been removed from the oocytes by brief treatment with hyaluronidase IV-S (Sigma-Aldrich) at 37°C. These desynchronized oocytes were incubated in Continuous Single Culture Complete medium (CSCM-C; Irvine Scientific) in an embryoscope (Geri time-lapse system, Genea Biomedx) at 37°C, 6% CO_2_ and 5% O_2_ to record the maturation process. *In vitro* maturation to the NEBD, metaphase I or metaphase II stages, activation or atresia were evaluated 24 h later. Oocytes were considered as ‘matured’ when the first polar body was present.

#### Additional datasets

##### Mouse datasets

To train our neural network for segmentation, we also used data from other projects to diversify our training. We used movies of mouse oocyte maturation acquired in transmitted light from a previous study (Bennabi et al., 2020). To train our neural network to determine the timing of NEBD, we also used another dataset of mouse maturing oocytes (C57BL6 strain) in which the NEBD timing had already been manually annotated (120 movies) (unpublished projects). To compare oocytes coming from two wild-type mouse strains, we added images of control oocytes (C57BL6) acquired before NEBD from other projects (157 images, unpublished projects) at a resolution of 0.1135 µm/pixel.

##### Human datasets

Through the ICSI procedure at the Cochin hospital (see above), we also had access to images of metaphase II oocytes that we used to diversify our training data (658 images). Moreover, we also added images from movies following the development of fertilized oocytes from the Hôpital Femme Mère Enfant in Lyon (551 movies). The cumulus–oocyte complexes (COCs) were obtained after transvaginal follicular puncture of patients treated for infertility.

One hour after COC retrieval, they were stripped by enzymatic digestion (hyaluronidase; CooperSurgical, Malov, Denmark) and by a mechanical action. The stripped oocytes were fertilized by intracytoplasmic sperm injection (ICSI). After ICSI, the fertilized oocytes were immediately cultured in oil-coated Cleav^®^ medium (CooperSurgical) in a time-lapse system (EmbryoScope®; Vitrolife, Viby, Denmark). The culture was monitored by successive image acquisitions (one image every 15 min).

##### Sea urchin dataset

Purple sea urchins (*Paracentrotus lividus*) were obtained from the Roscoff Marine station (France) and kept at 16°C in an aquarium for several weeks in artificial seawater (Reef Crystals; Instant Ocean). Gametes were collected by intracoelomic injection of 0.5 M KCl. Sperm was collected dry and kept at 4°C for 1 week. Eggs were rinsed twice, kept at 16°C, and used on the day of collection. The jelly coat of unfertilized eggs was removed by passing them three times through an 80-μm Nitex mesh (Genesee Scientific) to allow egg adhesion on protamine-coated glass-bottomed dishes (MatTek Corporation). Pictures were acquired before fertilization or 1–5 min after fertilization.

Image acquisition was performed on a wide-field microscope (TI-Eclipse; Nikon) equipped with a complementary metal oxide-semiconductor camera (Orca-flash4.0LT; Hamamatsu). Samples were imaged with a 20× dry objective (NA, 0.75; Apo; Nikon). A secondary microscope (Leica DMI 6000B) equipped with the same camera using either a 20× dry objective (NA, 0.70; PLAN Apo; Leica) or a 10× dry objective (NA, 0.25; PLAN; Leica) was also used. Microscopes were operated with Micro-Manager (Open Imaging). A total of 185 images were used for training and 50 were used for testing.

All animal studies were performed in accordance with the guidelines of the European Community and were approved by the French Ministry of Agriculture (authorization no. 75-1170) and by the Direction Générale de la Recherche et de l’Innovation (DGRI; GMO agreement number DUO-5291).

### Ground-truth database creation

Before training the neural networks, we tried to segment images using an approach based on thresholding and morphology. However, the results were highly dependent on the input parameters and could not be used without manual validation and correction. We therefore chose to use a deep learning approach instead to improve the performance. This preliminary segmentation allowed to build an initial database with the manually validated and corrected ground-truth segmentation. Moreover, in the additional dataset from a previous project (Bennabi et al., 2020), some oocytes were also stained with a fluorescent membrane marker, which allowed direct access to the ground-truth images for these oocytes. In the end, we created a database of 8256 images (4432 for mouse, 3824 for human) with their associated ground truth. This dataset is publicly available on Zenodo (Letort et al., 2022b), so that it can be used freely to train other neural networks.

Eventually, the creation of the ground truth for the detection of the zona pellucida was more challenging as the contrast was often very low. To counter this, the segmentation was done or corrected manually on a large part of the dataset by defining an ellipse around the zona pellucida and thus lacked precision. This could explain the lower performance of the neural network on zona pellucida segmentation against this imprecise ground truth. The database for the zona pellucida consisted of 3578 images (2361 for mouse, 1217 for human) with their associated ground truth.

It is important to note that multiple images per movie were used in the datasets to increase the number of images. However, when a dataset was split between training and test subsets, as well as when the training dataset was split for cross-validation, images extracted from the same movie were always in the same subset to ensure that we had independent datasets and avoid data leakage (Wen et al., 2020).

### Machine learning pipeline

#### Pipeline preprocessing

First, movies and/or images had to be preprocessed. When several oocytes were present in the same movie, they were automatically cropped into several image stacks of a single entire oocyte. Moreover, the movies were aligned to keep the oocyte at the same position in the images to measure dynamic features. Finally, intensity normalization (min-max normalization) was performed to homogenize the images coming from different sources.

#### Oocytor plugin

Oocytor is a Fiji plugin (Schindelin et al., 2012) implemented in Java. The source code can be found on GitHub (https://github.com/gletort/Oocytor), as well as a compiled version presented as a ready-to-use plugin, with installation instructions. Oocytor can perform three tasks: oocyte contour segmentation, NEBD detection and features extraction. Segmentation and NEBD detection are based on our neural network trained on large databases in Python. To run the already trained neural network in Fiji, Oocytor uses the CSBDeep plugin (http://sites.imagej.net/CSBDeep) (Weigert et al., 2018). To calculate some features, Oocytor uses FeatureJ (http://imagescience.org/meijering/software/featurej). We have also suggested using several macros in the GitHub repository to facilitate Oocytor usage with several data folders.

#### Oocytor NEBD detection

##### Neural network implementation and training

We built our neural network to classify images based on the presence or absence of a nucleus with the VGG-16 architecture (Simonyan and Zisserman, 2015 preprint) and tested variations around this architecture. The final architecture is shown in Fig. S1A. To train the neural network for NEBD detection, we used a one-time data augmentation on the training images, by flipping these images. Images were resized to 256×256 pixels and normalized before being fed to the neural network. We trained the neural networks for 25 epochs, with batch normalization and a batch size of 30, rectified linear unit (ReLU) activation functions and binary cross-entropy loss. To adjust the network hyper-parameters, we performed tenfold cross-validation on the training dataset (Fig. S1B, selection of the number of filters *n* in the initial layer). The selected network was then trained on the full training dataset and its performance was tested on the independent test dataset (Fig. 1C, right panel).

##### NEBD determination in Oocytor

Each image of the movie is resized to 256×256 pixels and normalized before running it through the neural network. This gives a score of the probability of the absence of the nucleus at each time point (Fig. 1D). The evolution of this score according to time is locally smoothed and NEBD is calculated as the first transition point from a low to high score (Fig. 1D, bottom left panel). This value of NEBD timing was then used to temporally align the results of the movies in the analysis.

#### Oocytor segmentation

##### Neural network implementation and training

We based our neural network architecture for cortex and zona pellucida segmentation on the U-Net architecture (Ronneberger et al., 2015). We tested several configurations around this architecture [number of layers, activation function, variation of the architecture (Falk et al., 2019; Gadosey et al., 2020; Ibtehaz and Rahman, 2020)] and opted for a classical U-Net architecture, shown in Fig. S1C. Note that we could have trained a single network to segment the zona pellucida and the cortex at the same time, which could have slightly improved the performance of the network (Firuzinia et al., 2021). However, we preferred to keep two independent networks for more flexibility. Images were resized to 256×256 pixels and normalized before being fed to the neural networks. We trained the neural networks for 25 epochs, with batch normalization and a batch size of 30, ReLU activation functions and jaccard distance loss. To adjust the network hyper-parameters (number of epochs, network parameters), we performed sixfold cross-validation on the training dataset (Fig. S1D,E, selection of the number of filters *n* in the initial layer). Based on these results, we selected a U-Net like architecture with *n*=8 initial filters for cortex segmentation (Fig. S1D) and with *n*=16 initial filters for zona pellucida segmentation (Fig. S1E). The selected networks were then trained on the full training dataset and their performance was tested on the independent test dataset (Fig. S1F).

##### Implementation of segmentation

Oocytor first resizes (to 256×256 pixels) and normalizes input images or movies. To increase the robustness of the plugin, the results of two neural networks trained on the same task (segmentation of oocyte contour or zona pellucida boundaries) were combined (Fig. S1G, ‘Get cortex’ function of Oocytor). The resulting binary images are converted into Fiji regions of interest (ROIs) that can eventually be refined to local variation of intensity or smoothed, depending on the input parameters of the plugin.

#### Features implemented

The complete list of features implemented in Oocytor, along with a brief description, is available on GitHub (https://github.com/gletort/Oocytor/blob/main/SupplementaryMaterials_features.pdf).

#### Feature selection

We calculated the Pearson correlation between all features (Fig. 2D) and set a threshold (0.7 here) above which features were considered correlated. By iterations, we kept the features with the most connections (correlated features) and removed those connected to it, until we obtained a subset of uncorrelated features that were below our threshold (Fig. 2D, top).

##### Feature standardization

Standardization was performed to shift the values of features in a similar range for all features. For each feature, the mean and standard deviation (s.d.) were calculated from the training data. The feature values were then updated as: f=(f−mean)/s.d.

### Machine learning methods

After features extraction with Oocytor, machine learning analysis was performed with the R software (https://www.r-project.org/). We used the ‘randomForest’ and ‘e1071’ packages (Liaw and Wiener, 2002; https://rdrr.io/rforge/e1071/) for the classification algorithms tested. UMAP projections were calculated with the ‘umap’ package https://cran.r-project.org/web/packages/umap/vignettes/umap.html; McInnes et al., 2018. Finally, graphs were generated with the ‘ggplot2’ package (Wickham, 2016).

#### Classification methods

We tried three standard machine learning methods in the Analysis step: Naive Bayes classifier (Rish, 2001), Support Vector Machine (Vapnik et al., 1996) and random forest (Breiman, 2001). Each of these algorithms received as input the features describing one oocyte and classified them. We used cross-validation techniques to measure the performance of our training and to select the best method (Fig. S2B, Fig. S4A,B). The selected method was finally trained on all the training data and its performance was always tested on an independent dataset.

#### Data imbalance

Datasets were strongly imbalanced between oocytes that entered maturation or not (Fig. S4A, left) as well as between oocytes that matured correctly or not (Fig. S4B, left). As we were interested in building a tool that would discriminate them based on images and not by considering the frequency of each class, we equilibrated the training datasets and measured the balanced accuracy to score the classification (Fig. S3A, right; Fig. S3D, right). For this, we used the number of the smallest dataset for both classes in the classification algorithm (under-sampling). We also tried to balance the dataset by data augmentation (oversampling) of the smallest dataset with the SMOTE technique (Chawla et al., 2002), but did not obtain better results.

#### Performance score

The scores used to measure the performance of the algorithms were: *accuracy*=(*TP*+*TN*)/(*TP*+*TN*+*FP*+*FN*), recall or 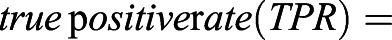 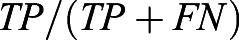, *precision*=*TP*/(*TP*+*FP*), 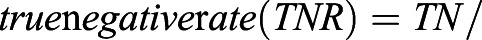 
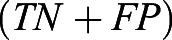
 and 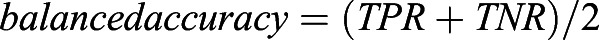,

where TP, TN, FP and FN correspond to true positive, true negative, false positive and false negative, respectively.

#### Comparison of human predictions with machine predictions

To compare the performance of our pipeline with manual prediction, we measured the performance of the pipeline on the new test dataset. We asked oocyte experts (people in our lab having a minimum of 5 years of expertise on mouse oocyte) to estimate the oocyte developmental potential from images taken 15 min before NEBD. Two newly appointed students, not fully trained yet, also participated (after a short training) in this contest, providing scores for a naive prediction (novice user).

#### Feature importance

To assess the contribution of each feature in the random forest algorithm, we considered the Gini index of the features in the decision trees. A higher Gini index indicates that the feature provides stronger separation of the population in the tree. Thus, features with higher Gini index were the most discriminant in the algorithm.

## Supplementary Material

Supplementary information
