# Supplementary material for: An interpretable and versatile machine learning approach for oocyte phenotyping
Source: J Cell Sci. 2022 Jul 13;135(13):jcs260281. doi: 10.1242/jcs.260281 (PMC9377708; doi:10.1242/jcs.260281)
Supplement: Supplementary information [file joces-135-260281-s1.pdf]

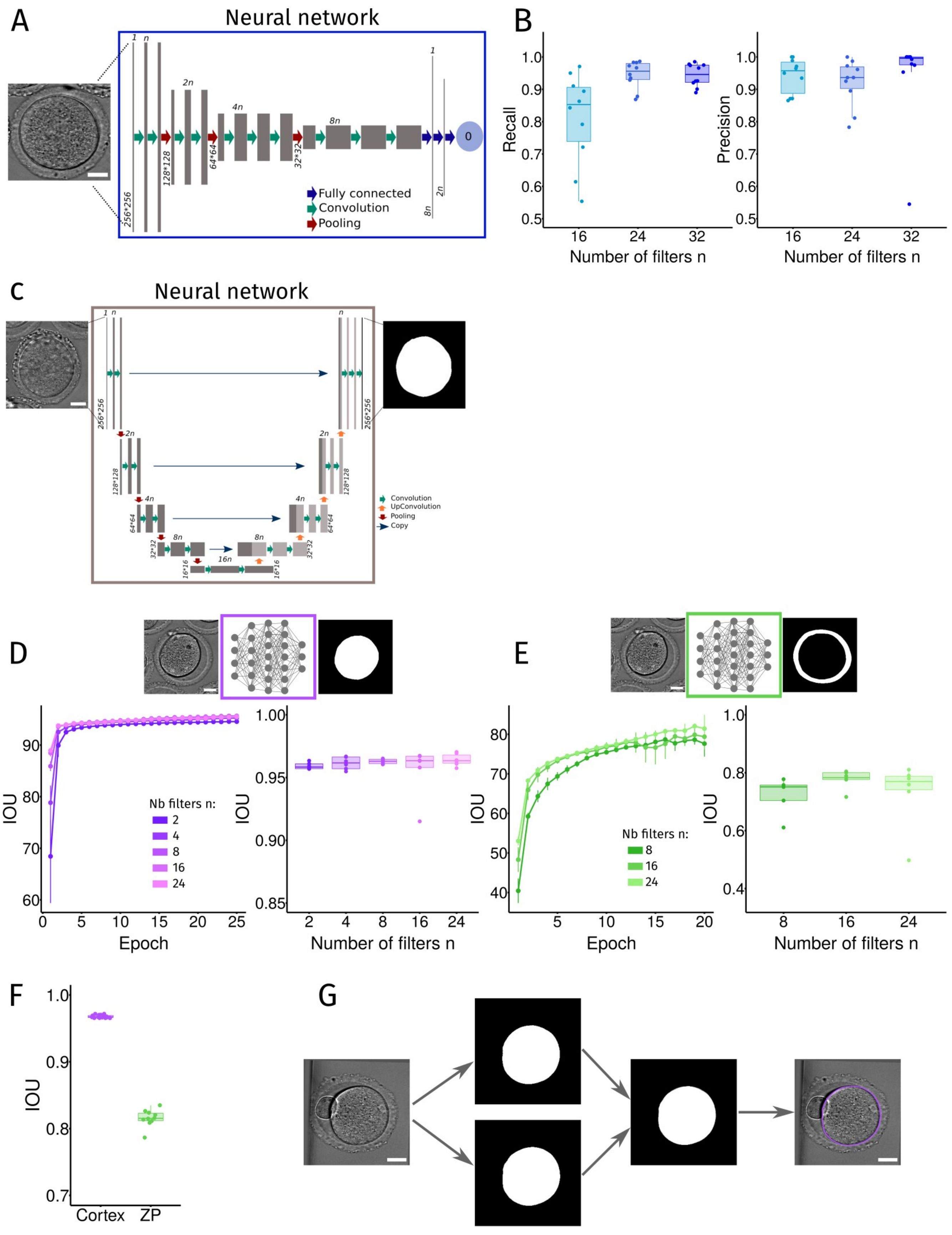

Fig S1

Journal of Cell Science • Supplementary information

Fig. S1. Neural networks for nucleus detection and oocyte contour segmentation.

A – Architecture of the selected neural network, based on the VGG-16 architecture. The network takes as input one image of an oocyte and gives as output a score of detection for the nucleus (0: presence of a nucleus, 1: absence). The first convolution layer applies (n) filters, which controls the size of the network and we tested several values for this parameter. Scale bar is 20 µm.

B – Score of nucleus detection from neural networks for different values of the number of initial filters (n) controlling the network size. The detection score was evaluated by Recall: TP/(TP+FN) and Precision: TP/(TP+FP), where TP=True Positive, FN=False Negative, FP=False Positive. The selection of the best parameter (n) was done with a 10-fold cross validation technique on the training dataset: data are split in 10 subsets, 9 are used for training, and the score is evaluated on the remaining one. The operation is repeated 10 times so that each subset is used as the test subset once. The final selected network had n=32 initial filters.

C – Architecture of the selected neural network, based on a U-Net architecture. The network takes as input one image resized to 256*256 pixels of an oocyte and outputs a binary mask with the interior of the oocyte. The size of the network is controlled by (n), the number of filters in the first convolution layer. Scale bar is 20 µm.

D – Selection of the proper neural network by cross-validation for oocyte membrane segmentation (purple box). The training dataset was split in 6 subsets for cross-validation. Networks with n=2, 4, 8, 16 or 24 initial filters were trained on 5 of the 6 subsets. Their performance during training was evaluated at each iteration (epoch) by the intersection over union (IOU) score between the network outputs and the ground truth images (left graph). The final performance of the trained network was validated on the respective remaining subsets by the IOU score (right graph). The network with n=8 filters was selected. Scale bar is 20 µm.

E - Selection of the proper neural network by cross-validation for zona pellucida contours segmentation (green box). By 6-fold cross-validation, the performance of neural networks with n=8, 16 or 24 initial filters (n) was evaluated during training by the IOU score (left graph) and after training on the remaining test subset (right graph). The network with n=16 filters was selected. Scale bar is 20 µm.

F – Performance of the neural network for membrane (purple dots) and zona pellucida (green dots) segmentation on independent test dataset for mouse oocytes measured by the IOU score.

G – Segmentation of the oocyte membrane with Oocytor. The input image is run through 2 neural networks trained on all our training dataset (mouse and human oocytes). Two networks were used to increase the robustness. Their outputs were combined in a final binary mask used to determine the oocyte contour. Scale bar is 20 µm. The procedure is the same in the case of zona pellucida segmentation.

Journal of Cell Science • Supplementary information

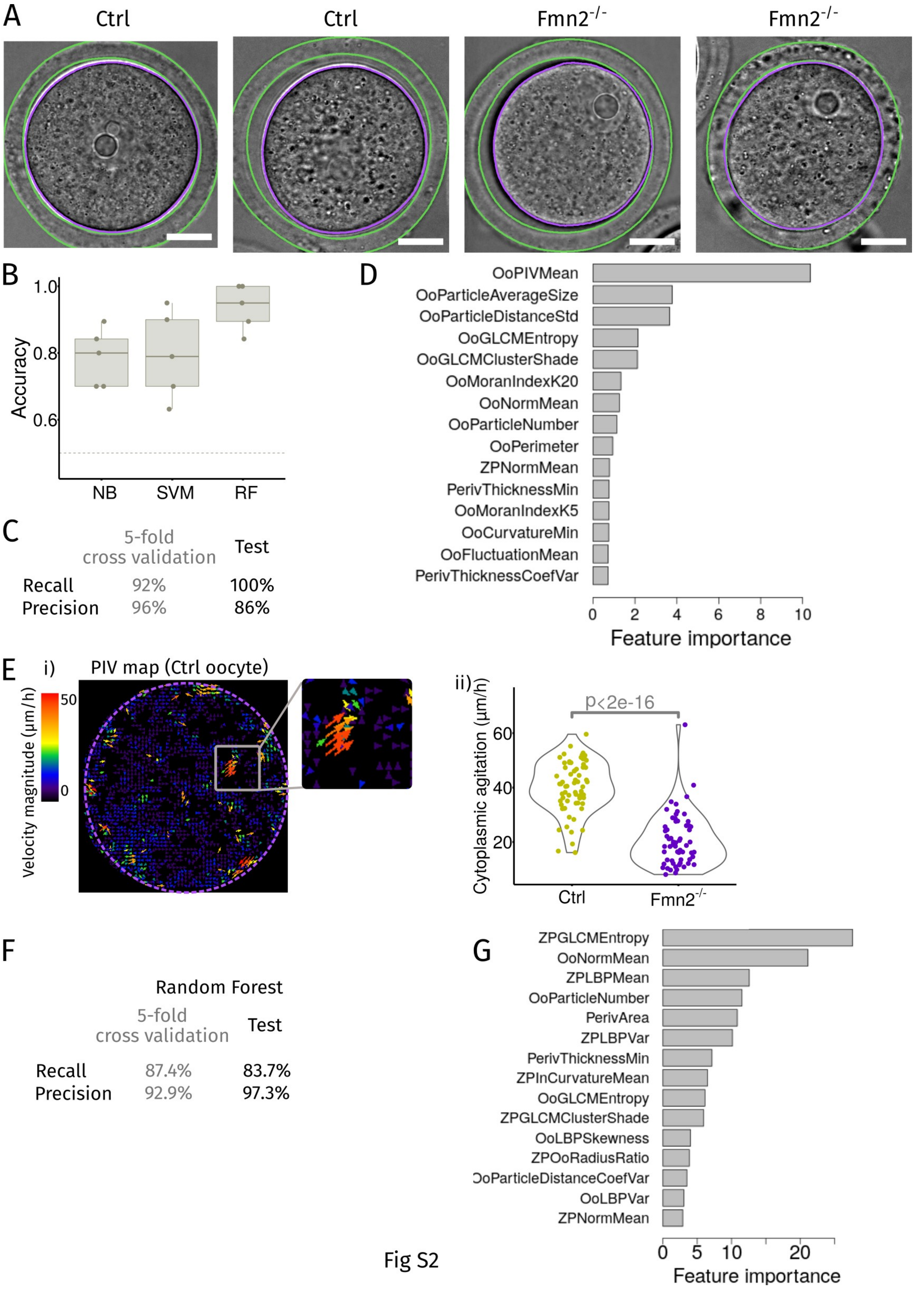

Fig S2

Journal of Cell Science • Supplementary information

Fig. S2. Phenotyping of oocyte populations using our machine learning pipeline.

A – Examples of segmentation of the oocyte contour (membrane, purple line and zona pellucida, green lines) obtained with our plugin on the new dataset. Scale bar is 20 µm.

B – Comparison of several classification algorithms performance. 3 methods were tested in the analysis step of our pipeline: Naive Bayes (NB), Support Vector Machine (SVM) and Random Forest (RF). Their performance was measured by their Accuracy and compared with a 5-fold cross-validation technique.

C – Scores of Random Forest classifier. Average prediction and recall scores are calculated on training data (by cross-validation) and test data.

D – Features classified by their importance using our algorithm. The features were sorted by their importance calculated from the Gini indexes of each features in our Random Forest algorithm. We only display the scores of the 15 most important features.

E – Cytoplasmic agitation appears as the most discriminant feature between Ctrl and $Fmn2^{-/-}$ oocytes. i) Example of a PIV (Particle Image Velocimetry) map extracted from the cytoplasm of a Ctrl oocyte: arrows indicate the direction of particle motion while their color reflects the magnitude of motion (purple, low to red, high). Insets correspond to a zoom of the image for better visualization. ii) Comparison of the mean value (average from all oocytes and from 5 min duration movies) of the cytoplasmic agitation, measured by the PIV in Ctrl (dark yellow) and $Fmn2^{-/-}$ (dark purple) oocytes. Statistical comparison was assessed using a Kolmogorov-Smirnov test (p-value indicated on the graph).

F – Scores of Random Forest classifier for the oocyte strain (OF1 vs C57BL6) phenotyping. Average prediction and recall scores are calculated on training data (by cross-validation) and test data.

G – Features classified by their importance using our algorithm to discriminate oocytes coming from two different wild-type strains. The features were sorted by their importance calculated from the Gini indexes of each feature in our Random Forest algorithm. We only display the scores of the 15 most important features.

Journal of Cell Science • Supplementary information

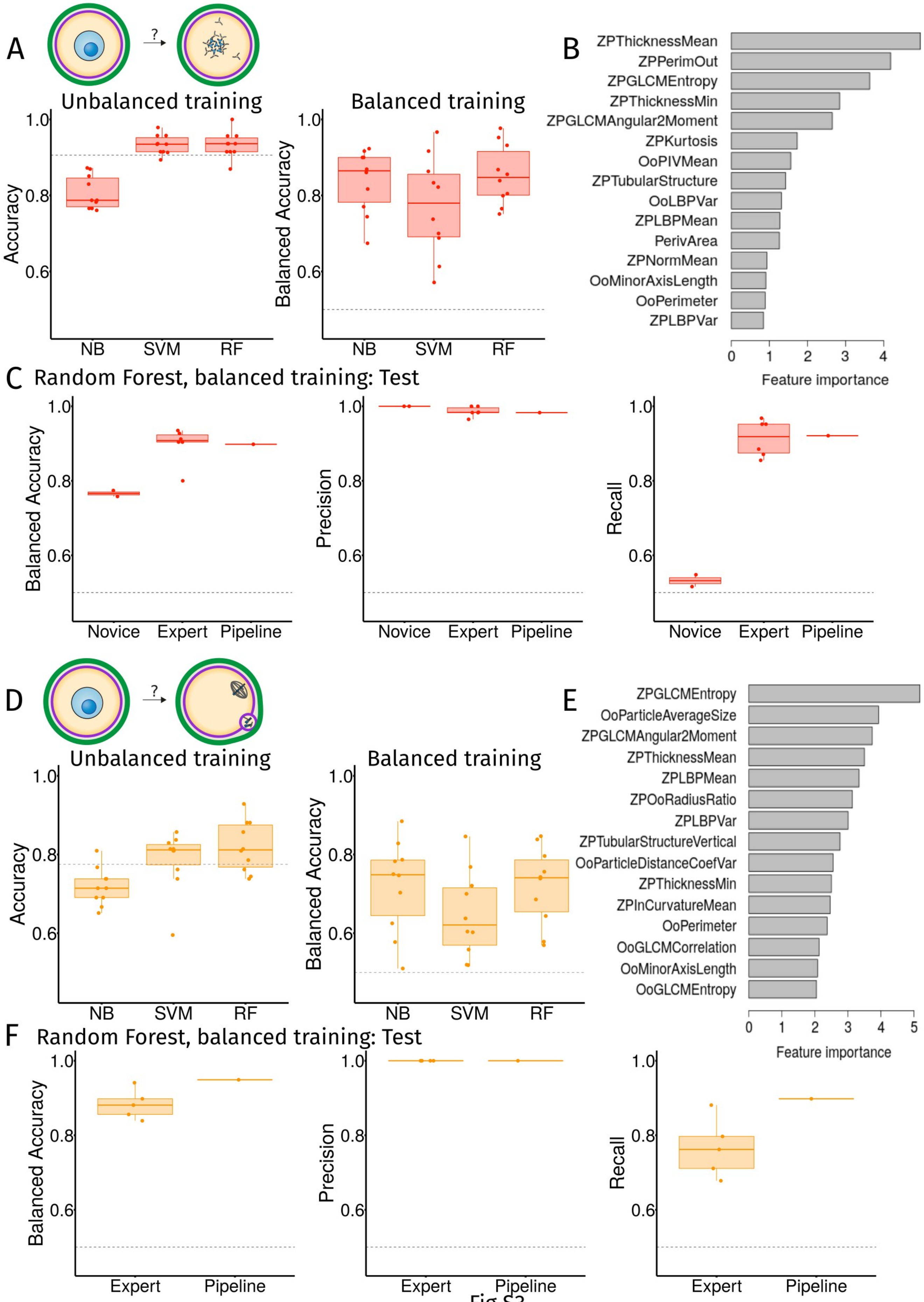

Fig S3

Journal of Cell Science • Supplementary information

Fig. S3. Prediction of NEBD failure and maturation defect.

A – Scores of prediction of NEBD failure with 3 classification methods (Naive Bayes NB, Support Vector Machine SVM, Random Forest RF) assessed by 10-fold cross-validation. The training was done with the unbalanced dataset, thus considering classes frequency, and the score was consequently measured by the Accuracy (left graph). Dashed line represents the naive prediction score consisting in always predicting NEBD success. The training was also done with under-sampling the over-represented class (middle graph) and the score was measured by the balanced accuracy. The dashed line represents the naive prediction.

B - Features classified by their importance for NEBD failure. Features sorted by importance calculated from the Gini indexes of each features in our Random Forest algorithm. We only display the scores of the 15 most important features. The description of features are given in Supplemental File 1.

C – Test on a new dataset of the selected algorithm (Random Forest on balanced training) performance (Balanced Accuracy, Precision, Recall), compared to the predictions of NEBD failure from novice and human experts. Balanced Accuracy is the average between the True Positive Rate and the True Negative Rate. Precision is sensitive to false positive: oocyte that will not resume meiosis but are predicted as correct. Recall is sensitive to false negative: oocyte that will resume meiosis but are not identified as correct.

D - Scores of predictions of maturation defect with the same 3 classification methods (NB, SVM, RF) assessed by 10-fold cross-validation. The training was done with the unbalanced dataset, thus considering classes frequency, and the score was consequently measured by the Accuracy (left graph). Dashed line represents the naive prediction score consisting in always predicting maturation success. The training was also done with under-sampling the over-represented class (middle graph) and the score was measured by the balanced accuracy. The dashed line represents the naive prediction.

E - Features classified by their importance maturation defect (right panel). Features sorted by importance calculated from the Gini indexes of each features in our Random Forest algorithm. We only display the scores of the 15 most important features. The significance of feature names are given in Supplemental File 1.

F - Test on a new dataset of the selected algorithm (Random Forest on balanced training) performance (Balanced Accuracy, Precision, Recall), compared to the predictions of maturation defect from human experts.

Journal of Cell Science • Supplementary information

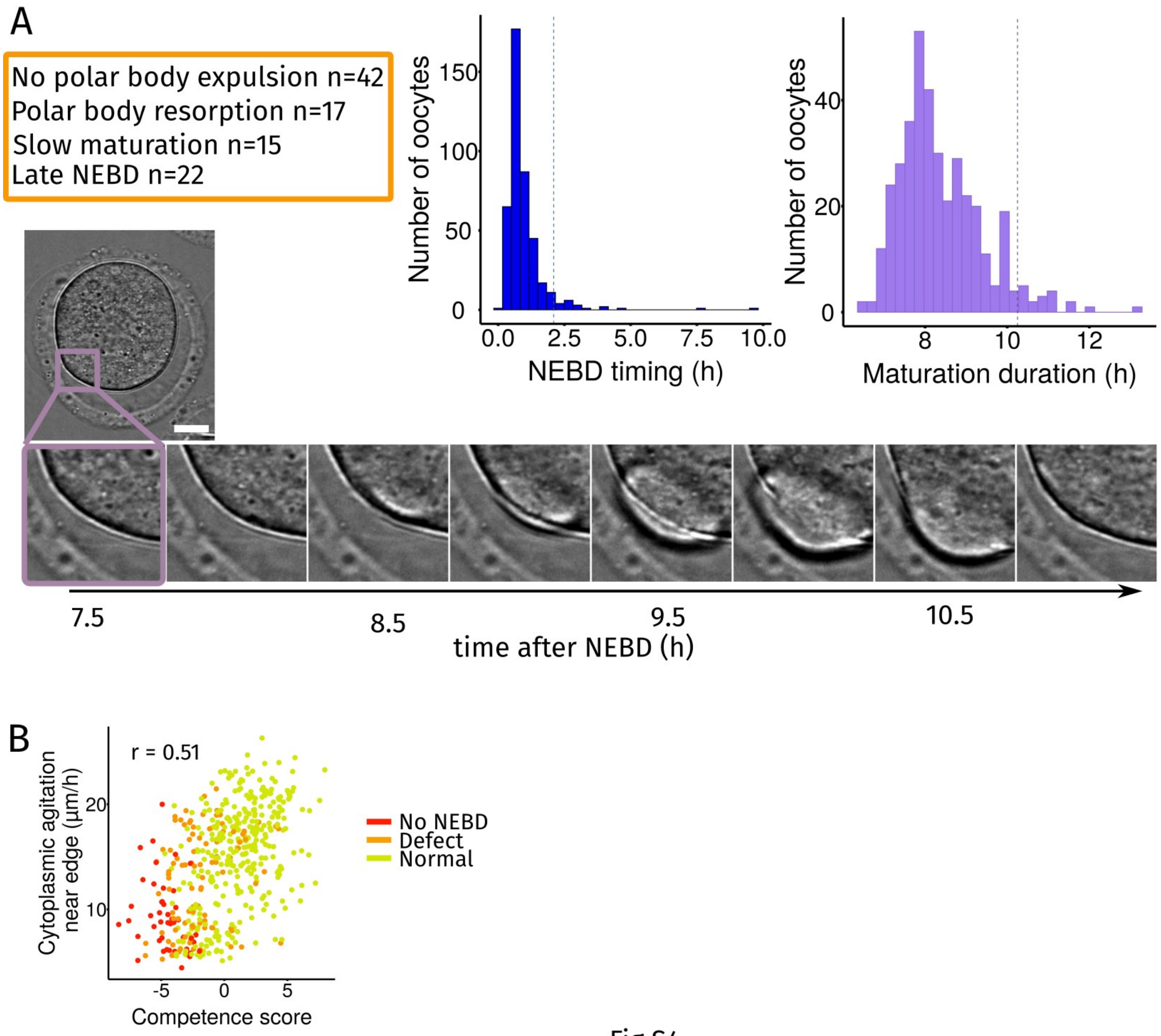

Fig S4

Journal of Cell Science • Supplementary information

Fig. S4. Characterization of oocyte maturation defects.

A – Criteria used to classify a maturation as defective (top left panel, n is the number of oocytes for each defect). Histogram of NEBD timing in our dataset (middle panel). The dashed line represents the 95% quantile after which oocytes are considered as delayed in NEBD timing. Histogram of the time between NEBD and first polar body extrusion in our dataset (right panel). The dashed line represents the 95% quantile after which oocytes are considered to have a slow maturation. Time lapse of an example of polar body resorption (bottom panel). Scale bar is 20 μm.

B – Correlation between cytoplasmic agitation and competence score. Cytoplasmic agitation in the area close to the edge of the oocyte (right panel) plotted against the oocyte competence score, for oocytes that do not enter into meiosis I (red, No NEBD), have a maturation defect (orange, Defect) or are normal (green, Normal). The Pearson correlation coefficients, r, are indicated in each graph with p-value < 10^-16 (Pearson’s product moment correlation coefficient) in both cases.

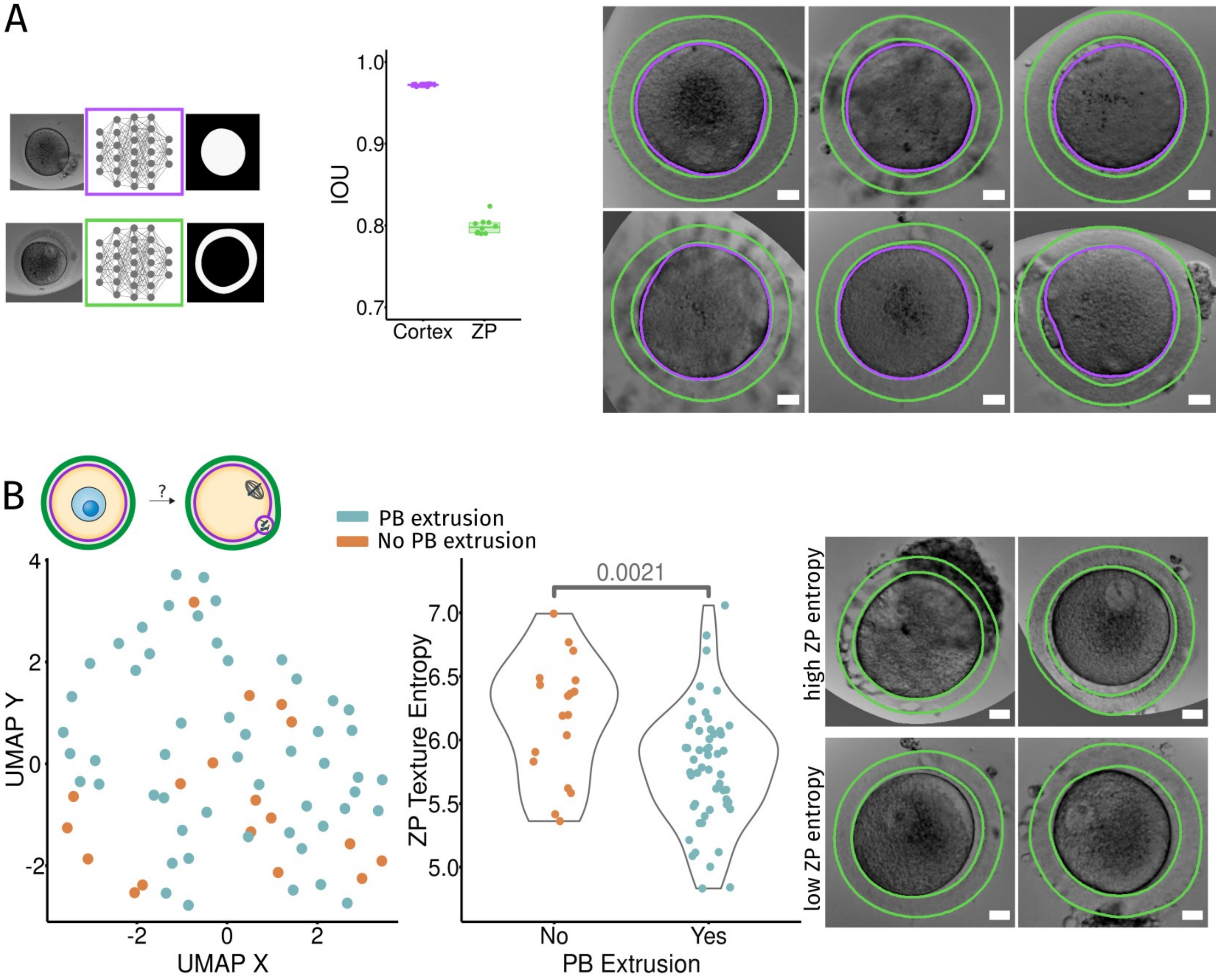
A
1.0
0.9
0.8
0.7
IOU
Cortex
ZP
B
?
PB extrusion
No PB extrusion
4
2
0
-2
UMAP Y
-2
0
2
UMAP X
0.0021
7.0
6.5
6.0
5.5
5.0
ZP Texture Entropy
No
Yes
PB Extrusion
high ZP entropy
low ZP entropy

Fig S5

Fig. S5. Characterization of human oocytes.

A – Segmentation of human oocyte membrane (purple box) and zona pellucida (green box) using our trained neural network (left panel). Graph of the score of the segmentation measured as the Intersection Over Union (IOU) on the human test dataset (middle panel). Examples of segmentation of human oocytes (right panel). The oocyte membrane appears as a purple line and of the zona pellucida contours as green lines after segmentation. Scale bars are 20 µm.

B – U-MAP projection of human oocyte features (left panel) for oocytes that will extrude a polar body (blue dots) and those who do not (orange dots). Values of the features at the beginning of the movies were used for all oocytes whether they had already entered meiosis or not. Comparison of zona pellucida texture entropy for oocytes that extrude a polar body or not (middle panel). Indicated p-value was calculated with a Kolmogorrov-Smirnov test. Examples of oocytes with high and low values for the entropy of the zona pellucida texture (right panel). Scale bars are 20 µm.
